# Aconserved salt-stress co-expression network in japonica rice reveals partially dissociated hub gene connectivity and transcriptional induction

**DOI:** 10.3389/fpls.2026.1914311

**Published:** 2026-09-14

**Authors:** Kun Xie, Song Hou, Na-Na Li, Min Liu, Fu-Gang Xin, Ru-Mei Tian, Jing Bai, Yun-Zhe Cong, Guan Li, Run-Fang Li, Han-Feng Ding, Yong-Yi Yang

**Affiliations:** National Medium-Term Genebank for Drought-Resistant and Saline-Alkali Tolerant Crop Germplasm Resources (Jinan), National Saline-Alkali Tolerant Crop Germplasm Resources Nursery (Dongying), Institute of Crop Germplasm Resources, Shandong Academy of Agricultural Sciences, Jinan, China

**Keywords:** co-expression network, hub gene, rice, RNA sequencing (RNA-Seq), salt stress, weighted gene co-expression network analysis (WGCNA)

## Abstract

**Introduction:**

Soil salinity severely compromises rice (*Oryza sativa* L.) growth and grain yield by disrupting ionic and osmotic homeostasis. Although transcriptome profiling has advanced understanding of salt-stress responses in rice, the conservation of co-expression networks across genetic backgrounds and the relationship between network connectivity and transcriptional induction remain poorly characterized.

**Methods:**

We performed integrated RNA sequencing and weighted gene co-expression network analysis (WGCNA) on three japonica rice varieties under control (0 mM), moderate (85.6 mM), and severe (136.9 mM) NaCl stress.

**Results:**

All three varieties exhibited basal salt tolerance. Differential expression analysis identified 2,067 and 4,900 non-redundant differentially expressed genes (DEGs) under moderate and severe stress, with 205 and 1,093 DEGs shared across the three varieties, respectively. WGCNA identified 32 co-expression modules. The brown module showed the strongest dose-dependent response to salt stress and was enriched in phenylpropanoid biosynthesis, plant hormone signal transduction, and MAPK signaling; its eigengene was highly concordant across varieties (mean r = 0.987), consistent with a shared leaf-level transcriptional program. Across brown-module genes, intramodular connectivity and salt-induced fold change were positively but only moderately correlated (Pearson r = 0.377): *OsDREB1A* was the most connected gene but not the most strongly induced, whereas *LOC_Os10g35080* was the most strongly induced yet ranked 14th in connectivity. In the 15-node hyperosmotic salinity response subnetwork, no single hub dominated, and *OsDREB1A* occupied a peripheral position.

**Discussion:**

Connectivity and induction thus reflect complementary aspects of the salt-stress response, and candidate hub genes for molecular breeding should be prioritized by both criteria jointly, followed by validation across diverse genetic backgrounds.

## 1 Introduction

Rice (*Oryza sativa* L.) is the staple food for more than half of the global population, and its production is increasingly threatened by soil salinization. According to the FAO’s 2024 global assessment, approximately 1.38 billion hectares of land are affected by salinity, accounting for 10.7% of the global land area (FAO, 2024). Around 10% of both irrigated and rainfed cropland are impacted, and salinity can reduce crop yields by up to 70% in severely affected regions (FAO, 2024). Salt stress impairs rice growth through two sequential phases: an early osmotic challenge caused by lowered external water potential and a later ionic phase in which excessive Na^+^ accumulation disrupts K^+^ nutrition and enzyme activity (Munns and Tester, 2008; Zhu, 2002). Germination, seedling growth and tillering are all suppressed under salinity, and grain yield declines accordingly (Li et al., 2024; Sackey et al., 2025). At the leaf level, salinity lowers leaf water status and induces stomatal closure (Munns and Tester, 2008; Sackey et al., 2025), while chlorophyll degradation and impaired PSII activity limit photosynthesis (Li et al., 2026). Impaired photosynthetic electron transport further promotes the accumulation of reactive oxygen species (ROS), which cause membrane lipid peroxidation and oxidative damage to cellular structures (Mittler, 2002). In response, plants activate osmotic adjustment, Na^+^ exclusion and vacuolar compartmentation, and enzymatic antioxidant defense systems (Munns and Tester, 2008; Sackey et al., 2025).

Variation in salt tolerance exists among rice germplasm. Salt-tolerant varieties maintain relatively normal growth and ion homeostasis under saline conditions, whereas sensitive genotypes exhibit severe growth inhibition and leaf chlorosis (Munns and Tester, 2008). The molecular basis of this divergence involves multiple regulatory layers, including the Salt Overly Sensitive (SOS) pathway for Na^+^/K^+^ homeostasis (Zhu, 2002; van Zelm et al., 2020), ABA-dependent and ABA-independent signaling, MAPK pathways, and transcriptional reprogramming by NAC, bZIP, and WRKY transcription factors (Zhu, 2002). Most of these studies, however, have centered on individual genes or pathways, including *OsSRO1c* in oxidative stress tolerance (You et al., 2013a), the *OsNAC2* –*OsAP37* module in salt-induced cell death (Mao et al., 2018), and the receptor-like cytoplasmic kinase STRK1 in H_2_O_2_ homeostasis (Zhou et al., 2018). How these processes are coordinated at the network level during salt stress poorly understood.

RNA-Seq captures genome-wide transcriptional changes under stress (Stark et al., 2019). WGCNA takes a systems-level view, grouping co-expressed genes into stress-responsive modules and pinpointing their hub genes (Langfelder and Horvath, 2008; Zhang and Horvath, 2005). In rice, this combination has yielded hub genes for heat stress (Wang et al., 2022) and anaerobic germination tolerance (Li et al., 2023). It has also been used to resolve gene regulatory networks and key genes underlying source–sink strength in cultivated and wild rice (Singh et al., 2025). In the WGCNA framework, a gene’s intramodular connectivity, quantified as module membership (MM), and its association with the trait of interest, quantified as gene significance (GS), are defined as distinct measures whose correlation varies across modules (Horvath and Dong, 2008). In a meta-analysis of abiotic-stress transcriptomes in cucumber, MM–GS correlations across stress-related modules ranged from 0.49 to 0.79 (Zinati and Nazari, 2023), indicating that intramodular connectivity and trait association, though related, are far from perfectly coupled. Candidate hub genes are therefore best prioritized by jointly applying both criteria rather than relying on a single measure. WGCNA-based salt-stress studies in rice have integrated transcriptomes across genotypes or compared two contrasting varieties (Zhu et al., 2019; Duan et al., 2024), but these analyses relied on pooled datasets or single pairwise comparisons, and the extent to which co-expression modules are conserved across genetic backgrounds remains unclear. Whether intramodular connectivity predicts the magnitude of transcriptional induction remains unknown in salt-stressed rice. This gap limits the cross-validation of connectivity-based hub gene prioritization. In this study, we subjected three japonica rice varieties–WYJ 31, LJ 11, and XN 8333–to control (0 mM), moderate (85.6 mM), and severe (136.9 mM) NaCl stress. Through comparative transcriptomics and WGCNA, we aimed to identify salt-responsive modules shared across genetic backgrounds, annotate the functional pathways within the module most strongly correlated with salt stress, and characterize the relationship between network connectivity and transcriptional induction among hub genes. In this study, we constructed a leaf-level salt-responsive co-expression network in japonica rice and prioritized candidate hub genes by jointly considering network connectivity and stress induction. These genes represent candidate targets for the molecular breeding of salt-tolerant rice.

## 2 Materials and methods

### 2.1 Salt stress treatment and sampling

Three japonica rice varieties with basal salt tolerance, WYJ 31, LJ 11, and XN 8333, were used in this study. Seeds were surface-sterilized with 75% ethanol for 1 min and 2.5% sodium hypochlorite for 15 min, then rinsed five times with sterile distilled water. After pre-germination, seeds were sown in hydroponic containers and cultivated in a climate-controlled growth chamber at 28 °C with a 14 h light/10 h dark photoperiod and 70–80% relative humidity. Seedlings were grown in Yoshida nutrient solution renewed every 3 days.

Salt stress treatment was initiated at the three-leaf stage, the developmental phase at which rice is most sensitive to salinity (Li et al., 2026). Three treatments were established. Control (CK), Yoshida nutrient solution without NaCl (0 mM); moderate salt stress (S5), nutrient solution supplemented with 85.6 mM NaCl (~5 g/L); severe salt stress (S8), nutrient solution supplemented with 136.9 mM NaCl (~8 g/L). Seedlings were exposed to two NaCl levels chosen from a recent dose–response study in japonica rice, in which growth was markedly inhibited at 90 mM whereas 170 mM was lethal within 10 days (Li et al., 2026). The lower level (85.6 mM) lies just below this critical concentration, and the higher level (136.9 mM) remains well below the lethal threshold and is comparable to the 130–140 mM NaCl used in previous studies of rice seedlings (Kim et al., 2005; Wang et al., 2016). The two levels thus imposed graded stress without causing seedling death, allowing transcriptional responses to be compared across stress intensities; no mortality was observed during the treatment (see Results). Each variety was subjected to all three treatments with three biological replicates (10 plants per replicate). The complete treatment timeline was as follows: pre-germinated seeds were grown hydroponically to the three-leaf stage, NaCl was then added to the nutrient solution, and the treatment was maintained for 10 days. After 10 days of treatment, leaf samples were collected, immediately frozen in liquid nitrogen, and stored at −80 °C until RNA extraction. A total of 27 samples were prepared for transcriptome sequencing.

### 2.2 RNA extraction and transcriptome sequencing

Total RNA was extracted using TRIzol reagent (Thermo Fisher Scientific, Waltham, MA, USA) and treated with DNase I to remove genomic DNA contamination. RNA integrity was assessed using an Agilent 2100 Bioanalyzer, and only samples with an RNA Integrity Number (RIN) > 8.0 were used for library construction. mRNA was enriched with oligo(dT) magnetic beads, fragmented, and reverse-transcribed into cDNA. Paired-end sequencing (2 × 150 bp) was performed on the Illumina NovaSeq 6000 platform (Illumina, San Diego, CA, USA), generating at least 6 Gb of clean data per sample. Library construction and sequencing were outsourced to BGI Genomics Co., Ltd. (Shenzhen, China).

### 2.3 Quality control, read alignment, and quantification

Raw reads were processed using fastp (v0.23.2) (Chen et al., 2018) for adapter trimming and quality filtering. Reads containing more than three ambiguous bases (N), reads shorter than 60 bp after trimming, or low-quality reads with Phred score < 20 in the sliding window analysis were removed. The clean reads were mapped to the *Oryza sativa* reference genome MSU7 (Kawahara et al., 2013) using HISAT2 (v2.2.1) (Kim et al., 2019) with default parameters. Transcript assembly and expression quantification were performed using StringTie (v2.1.7) (Pertea et al., 2015), and TPM and FPKM values were calculated for each gene.

### 2.4 Differential expression analysis

Differential expression analysis was performed using DESeq2 (v1.42.0) (Love et al., 2014). Differentially expressed genes (DEGs) between each salt treatment and the corresponding control were identified for each variety using the criteria of |log_2_FoldChange| ≥1 and adjusted *P*-value (*p*.adj) < 0.05. DEGs were classified as upregulated or downregulated, and Venn diagrams were used to compare shared and variety-specific DEGs among the three varieties. Volcano plots and heatmaps were generated for visualization.

### 2.5 Functional enrichment analysis

GO and KEGG pathway enrichment analyses of the DEGs were conducted using clusterProfiler (v4.0) (Yu et al., 2012). Gene annotations were retrieved from the org.Os.eg.db package (GO) and the KEGG database (Kanehisa et al., 2017). Significantly enriched terms and pathways were identified using the hypergeometric test with Benjamini-Hochberg false discovery rate (FDR) correction. GO terms with an adjusted *P*-value < 0.01 were considered statistically significant, whereas KEGG pathways with an adjusted *P*-value < 0.05 were considered statistically significant.

### 2.6 Weighted gene co-expression network analysis

WGCNA was conducted using the WGCNA R package (v1.70-3) (Langfelder and Horvath, 2008). An unsigned similarity matrix was constructed by calculating the absolute Pearson correlation coefficients between all gene pairs across the 27 samples. The adjacency matrix was calculated by raising the similarity matrix to a power of β = 6, which was selected as the optimal soft-thresholding power based on a scale-free topology fit index (R^2^ > 0.85) and mean connectivity. The weighted adjacency matrix was converted into a topological overlap matrix (TOM) to measure gene interconnectedness, and hierarchical clustering was performed using TOM-based dissimilarity (1 − TOM) with the average linkage method. Modules were detected using the dynamic tree cut algorithm (minModuleSize = 30, deepSplit = 2). Highly similar modules (module eigengene correlation > 0.75) were merged at a cut height of 0.25. Each module was assigned a unique color for visualization. The grey module comprised genes that could not be assigned to any co-expression module and was excluded from subsequent analyses. Module eigengenes (MEs), defined as the first principal component of the standardized expression matrix of each module, were computed for all 27 samples using the moduleEigengenes() function. For each module and variety, ME values of the three biological replicates were averaged per treatment; a module was considered associated with severe salt stress when its mean ME was higher under S8 than under CK in all three varieties.

### 2.7 Module cross-variety concordance analysis

Standard WGCNA modulePreservation() analysis relies on Zsummary statistics that depend on sample size. Permutation-based significance estimates become unreliable when sample sizes are very small (Langfelder et al., 2011). With only nine samples per variety—below the minimum of 15 recommended for robust correlation network analysis—module preservation testing lacked sufficient statistical power. We therefore employed eigengene-based cross-variety concordance as an alternative validation.

### 2.8 Subnetwork selection criteria

Brown-module genes annotated to GO:0042538 (hyperosmotic salinity response) were identified from the GO enrichment analysis. Intramodular connectivity (kIM) was calculated for each gene using the intramodularConnectivity() function in the WGCNA R package. The brown-module edge list was simultaneously exported via exportNetworkToCytoscape() for network visualization. Within this pathway, genes ranking in the top 30% of kIM were designated hub genes, with a minimum of five genes. Six genes were annotated to GO:0042538, and the five with the highest kIM were selected. Because six genes were insufficient for network visualization, the gene set was expanded to 15 nodes. *LOC_Os01g51420*, the remaining pathway gene, was retained. Nine other brown-module genes with the highest summed edge weights to the five hub genes were added. These ten genes served as structural nodes. Edges were ranked by weight. Hub-structural edges above the weight threshold (38 edges) together with the top 25 structural-structural edges were retained, yielding 63 edges. The subnetwork was drawn in a circular layout with hub genes in the inner ring and structural genes in the outer ring, using NetworkX and Matplotlib in Python (v3.11). Node size was proportional to kIM.

## 3 Results

### 3.1 Phenotypic responses to salt stress

After 10 days of salt stress treatment, the three rice varieties showed varying degrees of growth inhibition. Under S5, seedlings of WYJ 31, LJ 11, and XN 8333 maintained relatively normal growth, with only slight leaf-tip chlorosis and curling compared with the CK. Under S8, more pronounced symptoms were observed, including leaf chlorosis, rolling, and growth retardation, although the plants remained viable. These observations indicate that the three varieties possess basal salt tolerance, supporting their suitability for comparative transcriptomic analysis under salt stress. Quantitative measurements supported these observations (**Table 1**). Plant height decreased by 17.0–20.9% under S5 and by 21.6–23.9% under S8 relative to CK. Total leaf number declined progressively with stress severity in all three varieties, and dead leaf number increased relative to CK. WYJ 31 retained the greatest total leaf number under both stress levels and the greatest plant height under S8. LJ 11 showed the largest height reduction under S8 (23.9%). Varietal ranking differed among the measured indices, however, indicating that phenotypic divergence among the three varieties was modest under the conditions tested. Biomass, root growth, tiller number, and chlorophyll content were not recorded in this experiment; we acknowledge this as a limitation and return to it in the Discussion.

**Table 1:** Quantitative phenotypic responses of three rice varieties after 10 days of salt stress (mean ± SD, three biological replicates).

Variety	Treatment	Height(cm)	Dead_leaves	Total_leaves
WYJ 31	CK	41.2 ± 4.8	2.0 ± 1.1	14.7 ± 4.1
WYJ 31	S5	32.6 ± 2.1	3.9 ± 0.8	8.7 ± 3.1
WYJ 31	S8	31.5 ± 2.4	5.2 ± 2.0	7.8 ± 3.4
LJ 11	CK	34.3 ± 2.9	2.0 ± 2.2	9.1 ± 2.6
LJ 11	S5	28.3 ± 3.1	3.4 ± 0.8	6.8 ± 2.0
LJ 11	S8	26.1 ± 2.2	3.3 ± 1.2	6.0 ± 1.2
XN 8333	CK	39.9 ± 4.2	1.5 ± 0.8	9.4 ± 2.7
XN 8333	S5	33.1 ± 3.6	3.5 ± 0.8	8.3 ± 1.9
XN 8333	S8	31.3 ± 2.0	3.3 ± 1.2	6.0 ± 1.7

### 3.2 RNA-Seq data quality assessment

Transcriptome sequencing of 27 rice leaf samples yielded 182.49 Gb of clean data after quality control, with an average of 6.76 Gb per sample. The Q30 percentage exceeded 86.84% for all samples. Alignment of clean reads to the *Oryza sativa* reference genome (MSU7) using HISAT2 yielded average mapping rates of 91.56% for WYJ 31, 95.44% for LJ 11, and 92.42% for XN 8333, indicating that the sequencing data were of high quality and suitable for downstream analysis (**Supplementary Table 1**).

Principal component analysis (PCA) was performed on the transcriptome data (**Figure 1**). PC1 and PC2 explained 7.8% and 6.1% of the total variance, respectively. Within each variety, treatment groups were ordered along PC1 by stress intensity (CK < S5 < S8), with the largest displacement in LJ 11; LJ 11 samples also had the highest PC1 scores under every treatment, so PC1 reflects both the treatment gradient and varietal divergence. Along PC2, CK samples of all three varieties scored consistently higher than their salt-treated counterparts, pointing to a shared stress response across genetic backgrounds. The low cumulative variance (13.9%) is expected given the multi-factorial design, in which variety, treatment, and their interaction all contribute to expression variation.

**Figure 1:**
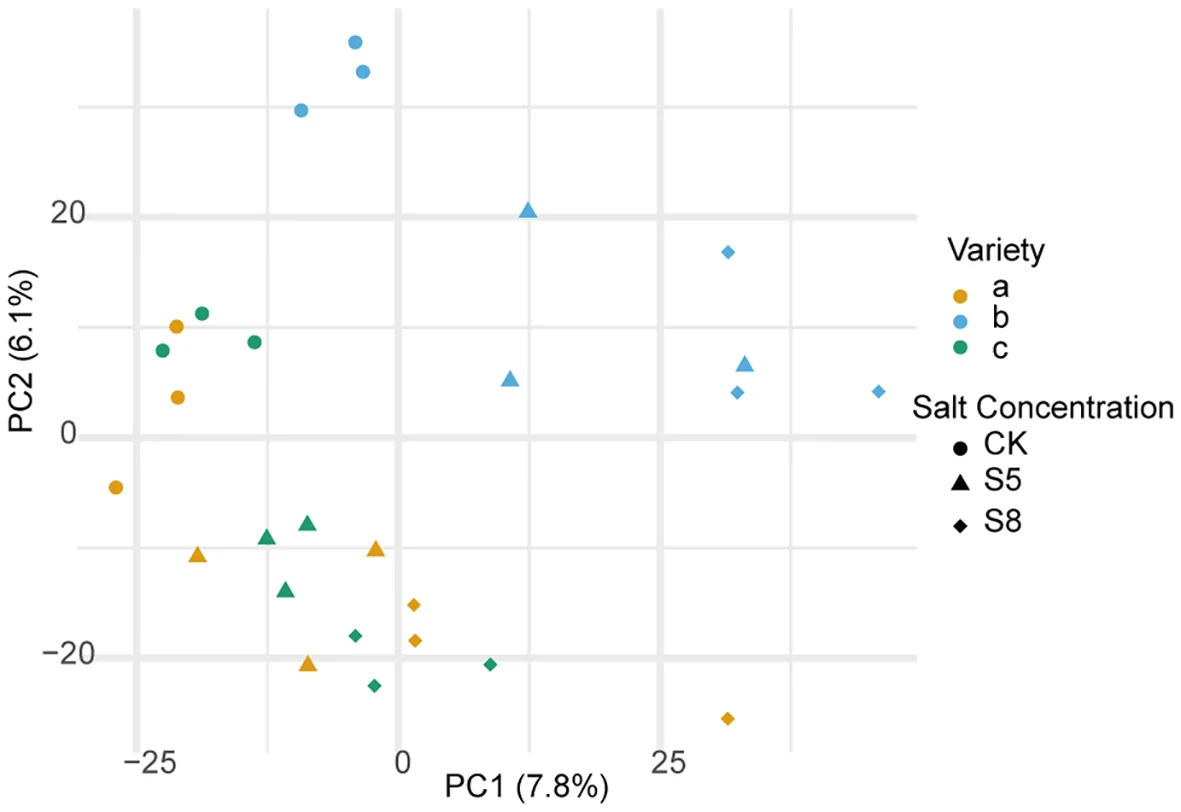
Principal component analysis (PCA) of transcriptome data from three rice varieties under different salt stress levels. Colors represent rice varieties: yellow = WYJ 31 **(a)**, blue = LJ 11 **(b)**, green = XN 8333 **(c)**. Shapes indicate salt treatments: circle = CK, triangle = S5, diamond = S8.

### 3.3 Transcriptome analysis of differentially expressed genes under salt stress

Per-variety differential expression analysis was performed comparing CK with S5 and S8. For CK vs. S5, WYJ 31 yielded 1,046 DEGs, LJ 11 yielded 3,523 DEGs, and XN 8333 yielded 1,383 DEGs; 205 DEGs were shared across all three varieties (**Supplementary Figure 1**). For CK vs. S8, WYJ 31 yielded 3,116 DEGs, LJ 11 yielded 7,283 DEGs, and XN 8333 yielded 3,951 DEGs; 1,093 DEGs were shared across all three varieties (**Supplementary Figure 2**). LJ 11 exhibited the strongest transcriptional response, consistent with its numerically largest height reduction under S8, while WYJ 31 showed the mildest response, consistent with its relative salt tolerance. Differential expression analysis on the pooled 27-sample dataset identified 2,067 and 4,900 non-redundant DEGs under S5 and S8, respectively.

Volcano plots visualized the global distribution of DEGs (**Figures 2A, B**). In both comparisons, upregulated genes were distributed on the right side (positive log_2_FoldChange values), whereas downregulated genes were distributed on the left (negative log_2_FoldChange values). The CK-vs.-S8 plot contained more statistically significant genes than CK-vs.-S5, reflecting the higher number of DEGs detected under severe salt stress.

**Figure 2:**
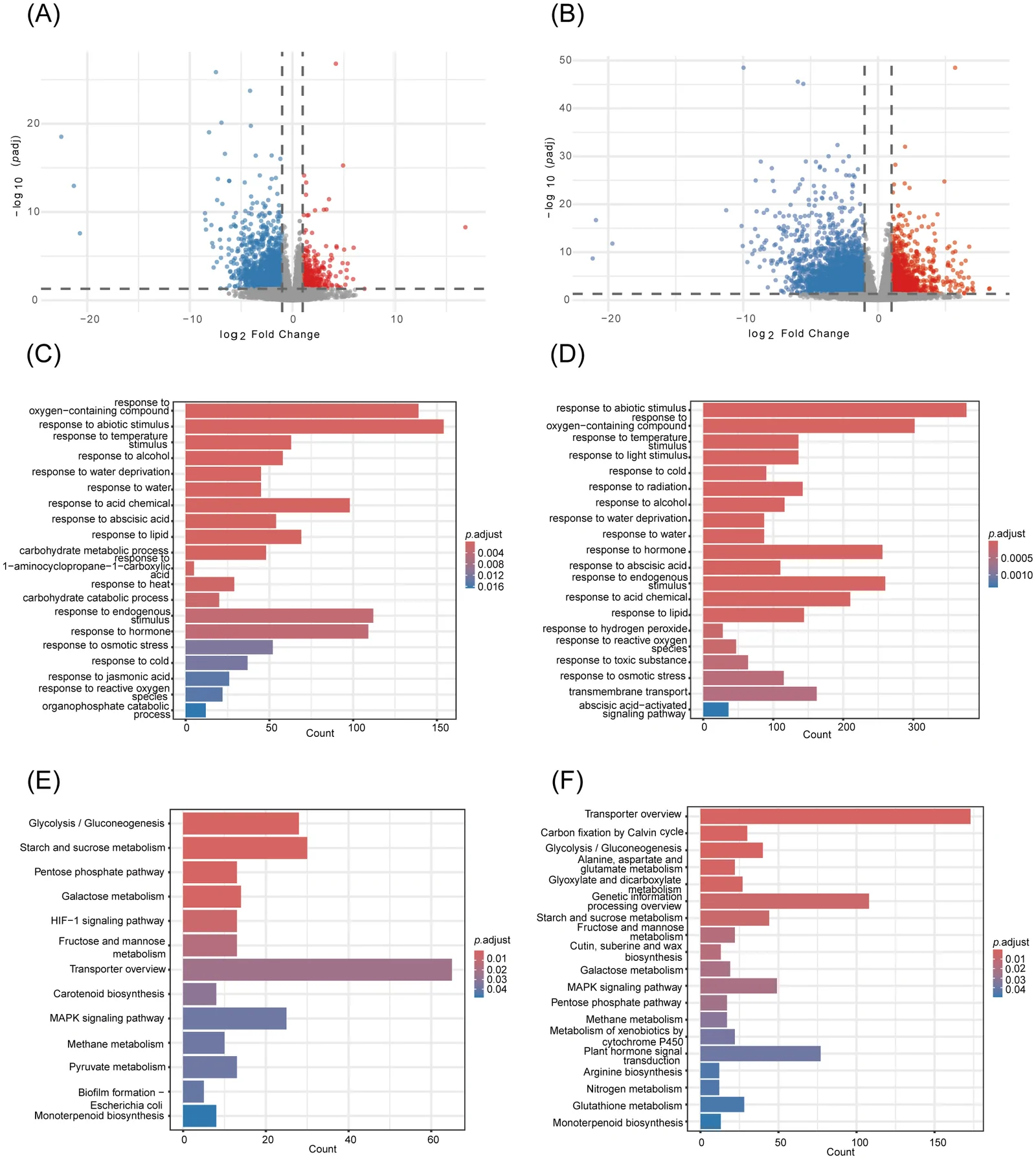
Differential gene expression and functional enrichment analyses under moderate and severe salt stress. **(A, B)** Volcano plots showing the distribution of DEGs in CK vs. S5 **(A)** and CK vs. S8 **(B)**. Red dots indicate up-regulated genes (log_2_FoldChange > 0), blue dots indicate down-regulated genes (log_2_FoldChange < 0), and grey dots represent non-significant genes. **(C, D)** GO biological process (BP) enrichment of shared DEGs under S5 and S8 salt stress. **(E, F)** KEGG pathway enrichment of shared DEGs under S5 **(E)** and S8 **(F)** salt stress. Bar colors indicate adjusted *P*-value levels.

GO biological process (BP) enrichment analysis of DEGs shared by all three varieties identified 36 and 57 significantly enriched terms under S5 and S8 salt stress, respectively (adjusted *P* < 0.01) (**Figures 2C, D**; **Supplementary Tables 2**, **3**). A total of 32 terms were shared between both conditions, representing 88.9% of all terms enriched under moderate stress, indicating that the two stress intensities activate a largely conserved set of biological processes. The most significantly enriched term was “response to abiotic stimulus” (GO:0009628), followed by “response to oxygen-containing compound” (GO:1901700) and “response to water deprivation” (GO:0009414). Other shared terms encompassed abiotic and oxidative stress responses (including osmotic stress, salt stress, and ROS), hormone signaling (abscisic acid and hormone-mediated pathways), and carbohydrate metabolism. These results indicate broad activation of stress perception, signal transduction, and energy mobilization under both stress levels. In contrast, 25 GO terms were exclusively enriched under severe salt stress, primarily involving ion transport, cellular hormone responses, and developmental regulation, suggesting that severe stress elicits additional mechanisms including disrupted ion homeostasis and aggravated oxidative damage beyond the conserved response.

KEGG pathway enrichment analysis of the shared DEGs identified 13 and 19 significantly enriched terms under S5 and S8 salt stress, respectively (adjusted *P*-value < 0.05) (**Figures 2E, F**; **Supplementary Tables 4**, **5**). Nine pathways were co-enriched in both conditions, including five carbohydrate metabolism-related categories, indicating salt stress broadly affects energy metabolic processes. The transporter overview pathway showed the strongest response escalation, consistent with the known importance of ion transport systems, including Na^+^/H^+^ antiporters and K^+^ channels, in salt stress adaptation. The MAPK signaling pathway was enriched in both conditions, reflecting its role in phosphorylation-based stress signal transduction.

Ten pathways were uniquely enriched in S8, among which carbon fixation by the Calvin cycle, alanine/aspartate/glutamate metabolism, glutathione metabolism, plant hormone signal transduction, and cutin/suberine/wax biosynthesis are notable. Co-enrichment of amino acid metabolism and glutathione metabolism in S8 but not S5 aligns with the enhanced requirement for compatible solute synthesis and ROS scavenging at higher salinity levels. The substantial DEG recruitment to plant hormone signal transduction further indicates extensive hormonal network reprogramming under severe stress.

Overall, the total number of DEGs detected across the three varieties increased from 5,952 under S5 to 14,350 under S8, a 2.4-fold increase. Concurrently, the number of enriched KEGG pathways expanded from 13 to 19, pointing to a dose-dependent transcriptional response. Moderate stress mainly affects carbohydrate metabolism and ion transport. Severe stress additionally impacts photosynthesis, amino acid metabolism, antioxidant systems, and structural barrier formation, suggesting a possible shift in carbon-nitrogen metabolic balance under severe salt stress.

### 3.4 Construction of co-expression network and module identification

WGCNA was performed on all gene expression profiles with a soft-thresholding power β = 6 (scale-free R^2^ > 0.85), yielding a mean connectivity of 47.3 connections per gene.

Dynamic branch cutting of the gene dendrogram based on topological overlap matrix (TOM) dissimilarity identified 32 co-expression modules (**Figure 3A**), ranging in size from 87 genes (paleturquoise) to 1,458 genes (turquoise). The turquoise module was the largest, followed by the blue (1,287 genes) and brown (1,143 genes) modules. The grey module (n = 282) comprised unassigned genes and was excluded from subsequent analyses.

**Figure 3:**
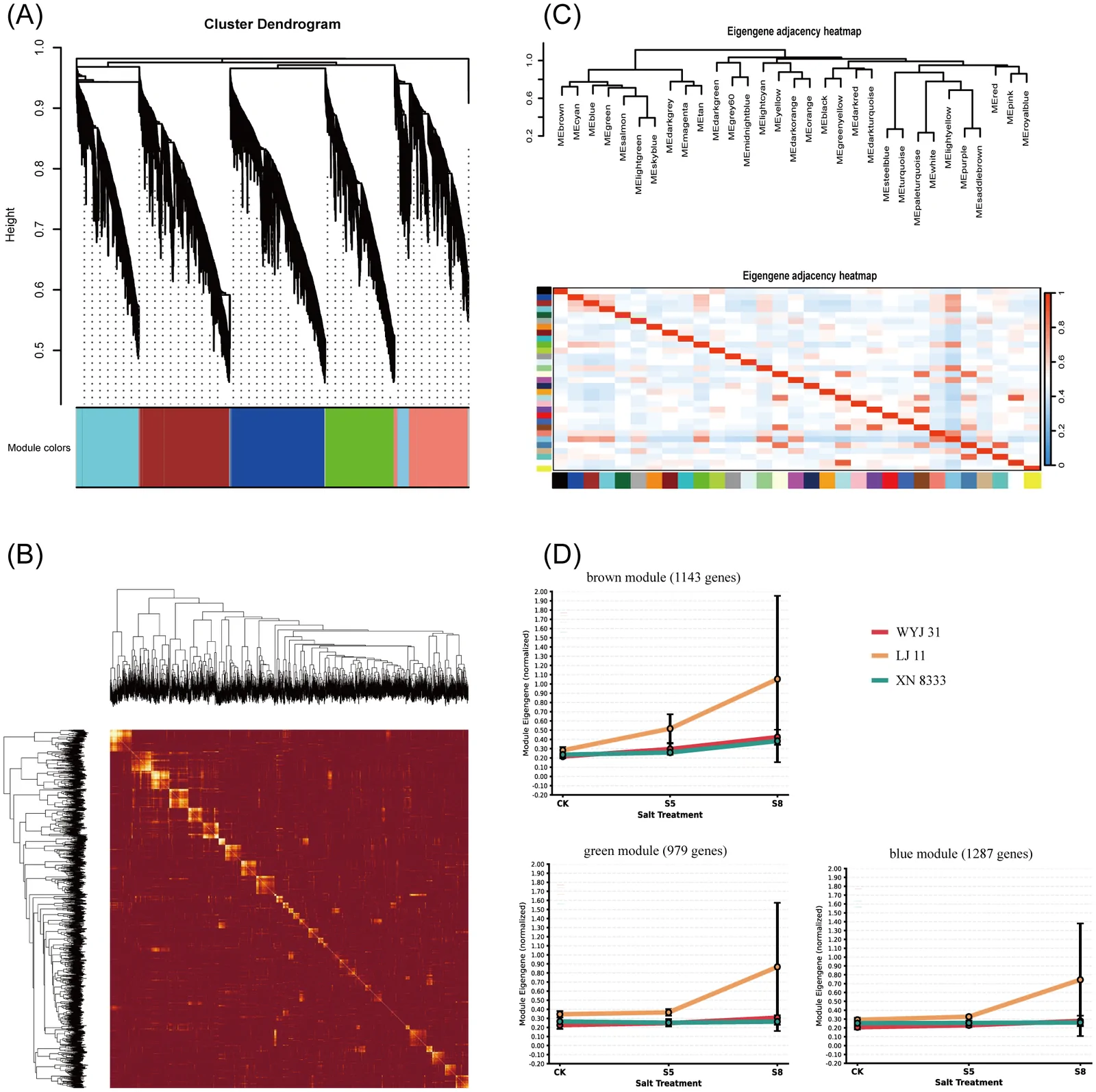
Weighted gene co-expression network analysis (WGCNA) of all genes under salt stress. **(A)** Gene dendrogram and module assignment by dynamic tree cutting (β = 6, scale-free R^2^ > 0.85). **(B)** Topological overlap matrix (TOM) heatmap for the top 2,000 genes, with hierarchical clustering dendrograms. Brighter (yellow) colors indicate higher topological overlap between gene pairs. **(C)** Module eigengene adjacency heatmap (bottom) and hierarchical clustering dendrogram (top) showing relationships among modules. Warmer colors represent higher adjacency. **(D)** Module eigengene (ME) values of the brown (1,143 genes), green (979 genes), and blue (1,287 genes) modules under salt treatments (CK, S5, S8) in WYJ 31, LJ 11, and XN 8333. Error bars indicate SD of biological replicates.

Network topology was validated by visualizing the TOM-based similarity matrix (top 2000 genes) as a heatmap superimposed with the gene dendrogram (**Figure 3B**). Diagonal blocks exhibited bright yellow-to-orange coloration, indicating high topological overlap among genes within the same module, whereas off-diagonal regions appeared dark red, reflecting low inter-module connectivity. This pattern supported that the module assignment captured biologically meaningful co-expression relationships.

Inter-module relationships were examined by calculating pairwise eigengene adjacencies. The eigengene adjacency heatmap displayed a prominent red diagonal, indicating strong intra-module cohesion (**Figure 3C**). Most off-diagonal elements were close to zero (blue/green), suggesting that the identified modules were largely independent transcriptional units. The turquoise and blue modules showed moderate positive adjacency, suggesting partial functional relatedness.

Module eigengenes (MEs) were calculated to characterize the expression patterns of each co-expression module across treatments (**Figure 3D**; **Supplementary Figure 3**). The brown, green, and blue modules showed higher ME values under salt treatment than under CK in all three varieties. In these modules, ME values increased progressively from CK to S5 and S8. This increase was most pronounced in LJ 11, whereas WYJ 31 and XN 8333 showed only moderate changes.

Because the network was constructed from the combined dataset, we next examined whether the modules behaved consistently in each genetic background. Module eigengenes computed separately per variety were highly correlated across varieties: for the brown module, pairwise correlations were r = 0.980 (WYJ 31 vs. LJ 11), r = 1.000 (WYJ 31 vs. XN 8333), and r = 0.980 (LJ 11 vs. XN 8333), with a mean of 0.987. The green and blue modules showed mean concordances of 0.997 and 0.996, respectively, and concordance values for all 32 modules are provided in **Supplementary Table 6**.

### 3.5 Functional enrichment of the brown module

GO biological process (BP) enrichment analysis showed that the brown module was associated with osmotic stress response. The most significant term was “response to water deprivation” (GO:0009414), reflecting activation of water-deficit-responsive mechanisms under salt stress (**Figure 4A**). “Regulation of seed germination” (GO:0010029) was also marginally significant, suggesting a possible link between salt-induced dormancy programs and the brown module, although this association requires further validation given the seedling-stage experimental design. Two oxidative stress-related terms, “response to reactive oxygen species” (GO:0000302) and “response to oxidative stress” (GO:0006979), reached nominal significance but did not survive FDR correction. This suggests that ROS-responsive genes in this module may operate in a cell-type- or condition-specific manner.

**Figure 4:**
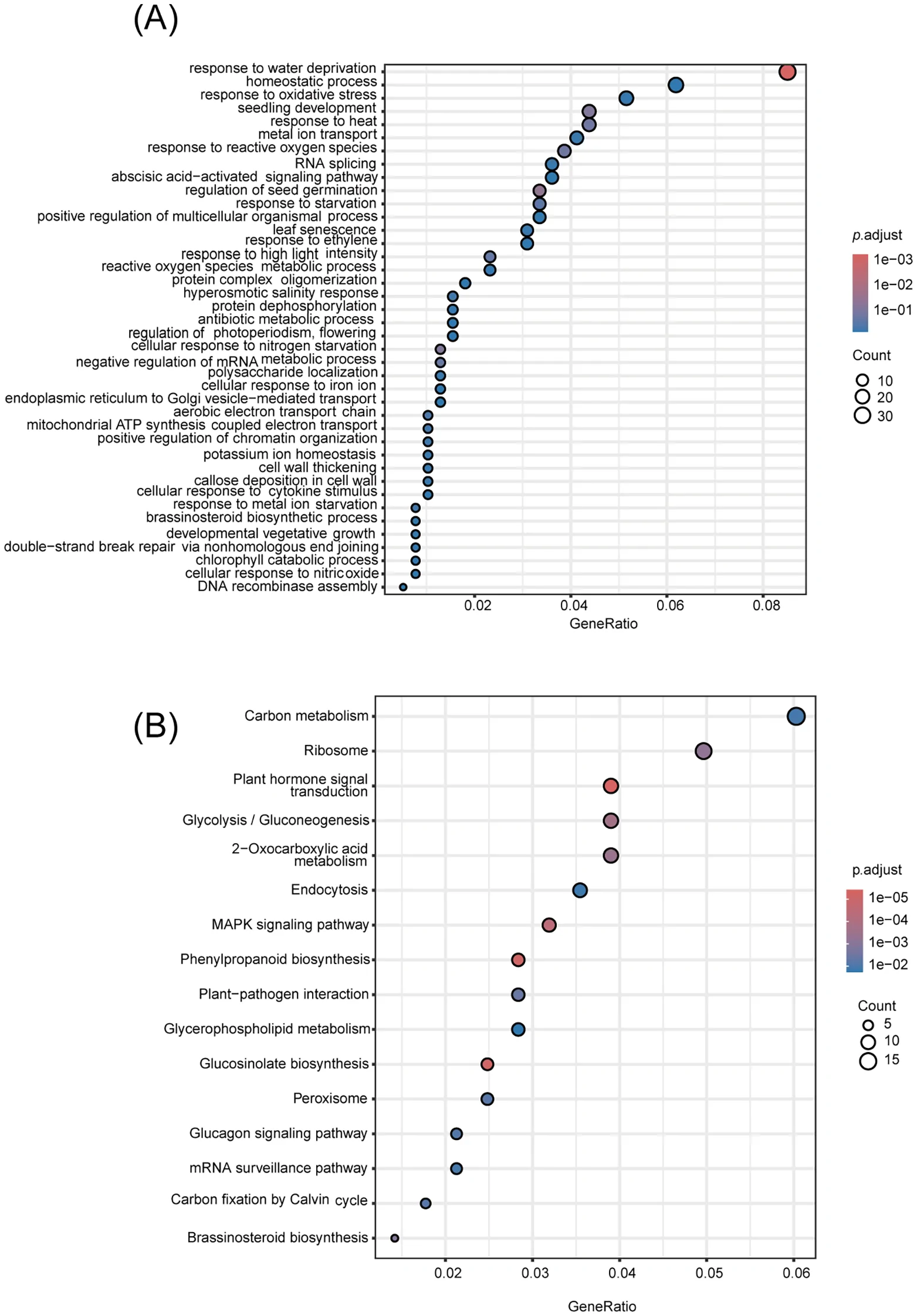
Functional enrichment analysis of brown module genes. **(A)** Top 30 enriched GO biological process (BP) terms ranked by adjusted *P*-value. **(B)** Top 15 enriched KEGG pathways. Bubble size represents gene count; bubble color indicates adjusted *P*-value (red = most significant, blue = least significant).

KEGG pathway analysis identified 16 enriched pathways (adjusted *P*-value < 0.05) (**Figure 4B**). Of these, seven pathways reached highly stringent significance (adjusted *P*-value < 0.001), spanning signal transduction (plant hormone and MAPK), secondary metabolism (phenylpropanoid and glucosinolate biosynthesis), and central carbon metabolism (glycolysis/gluconeogenesis and 2-oxocarboxylic acid metabolism), along with the translational machinery (ribosome). The most significant was “plant hormone signal transduction” (ko04075). Within this pathway, multiple hormone signaling branches together with Ca^2+^ signaling components were represented: ABA (ABF, SnRK2, PP2C), auxin (IAA, SAUR, GH3), ethylene (EIN2, EIN3), cytokinin (AHP), and Ca^2+^(calmodulin, CPK), indicating broad hormonal reprogramming under salt stress. “MAPK signaling pathway” (ko04016) was also enriched, containing MAPKKK17/18, OXI1, and the transcription factor VIP1. Two secondary metabolism pathways reached significance: “phenylpropanoid biosynthesis” (ko00940) and “glucosinolate biosynthesis” (ko00966). The seven glucosinolate biosynthesis genes were concurrently mapped to “2-oxocarboxylic acid metabolism” (ko01210), suggesting that this enrichment likely reflects amino acid metabolic flux rather than specialized glucosinolate production in rice. Multiple enzymes in the phenylpropanoid pathway (C4H, 4CL, CCR, CAD, POD) are consistent with enhanced flux toward monolignol biosynthesis, potentially contributing to cell wall reinforcement. Among energy metabolism pathways, “glycolysis/gluconeogenesis” (ko00010) showed the strongest enrichment, followed by “2-oxocarboxylic acid metabolism” (ko01210). The co-occurrence of ko01210 with both energy metabolism and glucosinolate biosynthesis annotations points to its role as a metabolic hub.

### 3.6 Hub gene identification and subnetwork analysis

The top 20 hub genes of the brown module, ranked by intramodular connectivity (kIM), showed high connectivity values ranging from 466.1 to 498.2 (**Table 2**; **Figure 5A**). All 20 genes were upregulated under S8, with fold change (S8/CK) values ranging from 5.1- to 203.2-fold. Eight genes (40%) showed moderate induction (FC < 25), including four with minimal response (FC < 10), while 12 genes showed strong induction (FC ≥ 25) (**Figure 5B**). Across all brown-module genes, kIM and fold change were positively but moderately correlated (Pearson r = 0.377, p = 7.2 × 10^-40^; Spearman ρ = 0.369, p = 3.2 × 10^-38^; **Supplementary Figure 4**), indicating that connectivity explains only part of the variation in transcriptional responsiveness (**Figure 5C**).

**Table 2:** Top 20 Hub Genes in the Brown Module Ranked by Intramodular Connectivity (kIM).

Rank	Gene (LOC_ID)	kIM	Mean CK (TPM)	Mean S8 (TPM)	FC (S8/CK)
1	LOC_Os06g45184	498.2	1.173	89.935	76.6
2	LOC_Os03g35650	490.8	0.013	1.039	78.8
3	LOC_Os01g20204	489.6	0.116	3.347	28.9
4	LOC_Os12g12080	489.3	1.150	63.490	55.2
5	LOC_Os03g05370	487.7	0.060	0.937	15.7
6	LOC_Os08g16910	487.5	0.014	1.346	98.8
7	LOC_Os01g36790	482.0	1.013	33.603	33.2
8	LOC_Os05g44030	481.9	0.193	6.300	32.6
9	LOC_Os07g47010	477.9	0.078	1.803	23.2
10	LOC_Os06g47720	477.9	0.235	1.230	5.2
11	LOC_Os09g24850	474.3	3.902	22.845	5.9
12	LOC_Os03g12820	472.2	37.675	667.603	17.7
13	LOC_Os09g03200	472.0	0.744	6.541	8.8
14	LOC_Os10g35080	470.9	0.028	5.786	203.2
15	LOC_Os04g37570	468.4	0.402	18.016	44.8
16	LOC_Os01g50900	468.2	0.048	1.977	41.5
17	LOC_Os06g05480	467.4	2.368	11.989	5.1
18	LOC_Os08g44360	467.3	0.036	3.124	87.5
19	LOC_Os04g58030	467.0	0.484	6.742	13.9
20	LOC_Os12g39330	466.1	0.024	2.519	104.4

FC values were calculated from unrounded mean TPM values.

**Figure 5:**
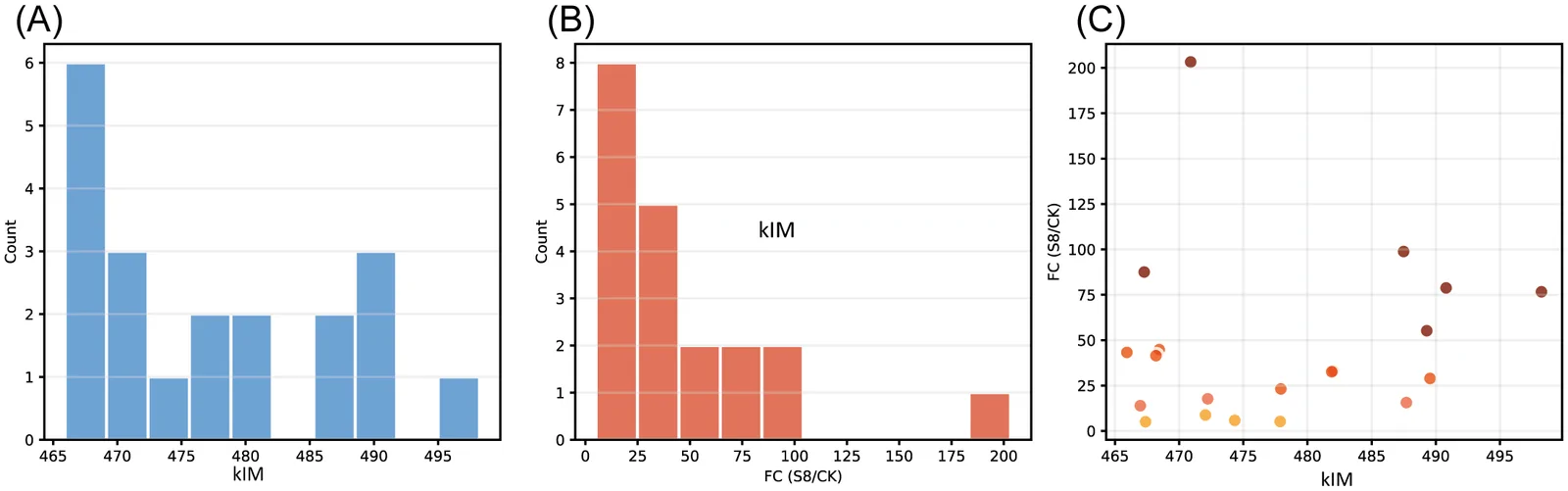
Network topology of the top 20 hub genes in the brown module ranked by intramodular connectivity (kIM). **(A)** Distribution histogram of kIM values. **(B)** Distribution histogram of fold change (FC, S8/CK). **(C)** Scatter plot of kIM versus FC. Color gradient from yellow to dark red indicates increasing FC magnitude.

Among these hub genes, *LOC_Os10g35080* (unknown function) showed the strongest induction. *LOC_Os06g45184* (*OsDREB1A*, AP2/ERF transcription factor), the most connected gene in the module, is a well-characterized abiotic stress-responsive transcription factor. *LOC_Os03g12820* (*OsSRO1c*) maintained high absolute expression in both control and salt stress conditions. *LOC_Os04g37570* (*OsAP37*, aspartyl protease) has been reported as a direct target of *OsNAC2* in salt-induced programmed cell death. *LOC_Os08g16910* (RLCK family protein kinase) was among the strongly induced hub genes.

Six brown-module genes were annotated to GO:0042538 (hyperosmotic salinity response). The five with the highest intramodular connectivity (kIM) — *LOC_Os01g62900*, *LOC_Os02g51290*, *LOC_Os07g05940*, *LOC_Os10g36180*, and *LOC_Os11g30500* — were designated hub genes. The remaining pathway gene, *LOC_Os01g51420*, together with nine brown-module genes strongly connected to the five hubs, served as structural nodes (**Figure 6**). The resulting subnetwork comprised 15 nodes and 63 edges. *LOC_Os06g45184* (*OsDREB1A*), the most connected gene in the brown module, occupied a peripheral position in this subnetwork. This distributed architecture, with no dominant central node, suggests coordinated action rather than control by a single hub gene in hyperosmotic salinity adaptation.

**Figure 6:**
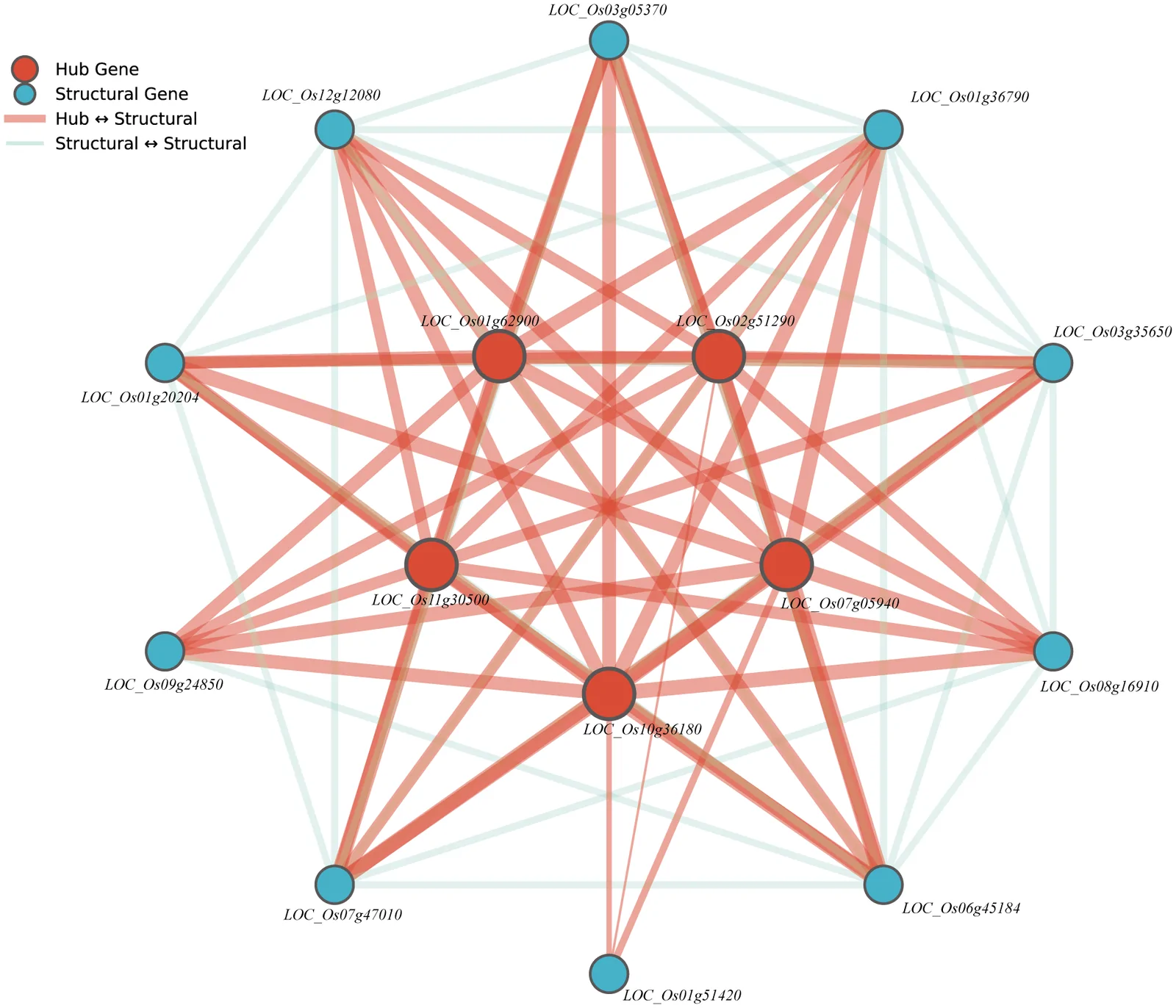
Co-expression subnetwork of the GO:0042538 (hyperosmotic salinity response) hub genes and their strongest co-expression partners in the brown module. Red nodes, five hub genes with the highest kIM among the six annotated genes (inner ring): *LOC_Os01g62900, LOC_Os02g51290, LOC_Os07g05940, LOC_Os10g36180*, and *LOC_Os11g30500*. Cyan nodes, ten structural genes (outer ring): the remaining pathway gene (*LOC_Os01g51420*) and nine genes selected by summed edge weight to the hub genes. Red edges, hub-to-structural links. Cyan edges, structural-to-structural links (top 25 by weight). Edge width indicates connection weight. Node size is proportional to intramodular connectivity (kIM).

## 4 Discussion

### 4.1 The brown module integrates osmotic signaling, structural defense, and energy metabolism

The brown module showed the strongest transcriptional response to severe salt stress across all three varieties. Pathway enrichment indicates statistical association, not direct functional evidence. The mechanistic interpretations below are therefore presented as working hypotheses awaiting experimental validation. Enrichment of “response to water deprivation” (GO:0009414) reflects the osmotic component of salt stress. Elevated Na^+^ lowers soil water potential around roots and reduces water uptake (Munns and Tester, 2008). The co-enrichment of phenylpropanoid biosynthesis (ko00940) and MAPK signaling (ko04016) points to coordination between rapid signal transduction and structural defense. Enzymes such as C4H, 4CL, CCR, and CAD channel flux toward monolignol production for lignin deposition and cell wall reinforcement (Vanholme et al., 2008). Lignin-modified cell walls may limit Na^+^ entry into the leaf apoplast under salinity, although ion fluxes were not measured directly. Root apoplastic barriers including Casparian bands and suberin lamellae are known to block Na^+^ transport to shoots (Krishnamurthy et al., 2011). The present transcriptome data were generated from leaf tissue. Any inference about functional coordination between leaf lignification and root barriers therefore remains hypothetical.

We focused on the brown module for three reasons, although the green and blue modules were also induced under S8. First, the brown module responded dose-dependently, with progressive eigengene elevation from CK to S5 to S8 (**Figure 3D**). Second, it showed the strongest induction under S8: the mean S8/CK eigengene fold-change was 2.54, compared with 1.72 for the green and 1.71 for the blue module (**Supplementary Figure 5**). Third, stress-adaptive pathways ranked among its most significant enrichment terms (adjusted P < 0.001), including plant hormone signal transduction, MAPK signaling, and phenylpropanoid biosynthesis. Basal-metabolism terms such as ribosome were also enriched in the brown module; however, the enrichment profiles of the green and blue modules were dominated by basal metabolic processes, such as ribosome and carbon metabolism (**Supplementary Tables 7**, **8**). High cross-variety concordance was not unique to the brown module (mean r = 0.987 for brown; 0.997 and 0.996 for the green and blue modules, respectively; **Supplementary Table 6**). The co-expression structure as a whole was therefore robust across genetic backgrounds.

The module was also enriched in plant hormone signal transduction (ko04075), with ABA signaling components (SnRK2, PP2C, ABF) as the most prominent. ABA-mediated phosphorylation cascades constitute a central regulatory axis for osmotic stress tolerance. PP2C phosphatases directly regulate SnRK2 kinases in this pathway (Umezawa et al., 2009), and three SnRK2 kinases function as the main positive regulators of ABA signaling under water stress (Fujita et al., 2009). Their enrichment is consistent with a prominent role of the brown module in salt stress adaptation. Salt stress simultaneously activated multiple hormone branches: auxin (IAA, SAUR, GH3), ethylene (EIN2, EIN3), and cytokinin (AHP) genes were all enriched, suggesting that salt stress triggers broad hormonal cross-talk rather than a single linear signaling cascade.

The enrichment of glycolysis/gluconeogenesis (ko00010) is consistent with enhanced glycolytic flux supporting ATP-dependent ion transport. The S8-specific enrichment of Calvin cycle genes further suggests that severe stress may impair photosynthetic carbon fixation; however, photosynthetic efficiency and chloroplast structure were not examined, and Na^+^/K^+^ contents were not measured, so these inferences await experimental verification. Metabolic reprogramming toward glycolysis may thus serve to maintain ATP supply to plasma membrane H^+^-ATPases and Na^+^/H^+^ antiporters under energy-limited conditions. Peroxidase (POD) genes within the phenylpropanoid pathway may serve a dual function: strengthening cell walls to limit Na+ apoplastic bypass while consuming H_2_O_2_ for ROS scavenging (Vanholme et al., 2008). The brown module also contained oxidative stress-related genes (GO:0000302; GO:0006979; uncorrected *P* < 0.001), although these terms did not reach significance after multiple testing correction. ROS-responsive genes in the module may operate in a cell-type- or condition-specific manner rather than as a uniform stress signature. Salt stress generates ROS through disruption of photosynthetic and respiratory electron transport, compounded by NADPH oxidase activation and diminished antioxidant capacity (Mittler, 2002). The variable representation of ROS-responsive genes in the brown module may mirror this condition-dependent oxidative burden.

### 4.2 Partial dissociation between connectivity and transcriptional responsiveness in hub genes

Intramodular connectivity and fold change were positively but only moderately correlated in the brown module (Pearson r = 0.377; Spearman ρ = 0.369). Thus, a gene’s network centrality did not predict the magnitude of its salt-induced expression change. This pattern parallels the moderate module membership–gene significance correlations reported in plant stress co-expression networks (Zinati and Nazari, 2023). It is also consistent with the WGCNA framework, in which connectivity and trait association are treated as independent dimensions (Langfelder and Horvath, 2008). Because the two measures were only partially coupled in this module, hub candidates were re-evaluated by both connectivity and fold change. *LOC_Os06g45184* (*OsDREB1A*) was the most connected gene in the module, but it was not the most strongly induced and occupied a peripheral position in the 15-node hyperosmotic salinity response subnetwork. *LOC_Os10g35080* (unknown function) was the most strongly induced hub gene but ranked 14th in connectivity. Its co-expression with brown module genes involved in phenylpropanoid biosynthesis, MAPK signaling, and hormone transduction suggests a potential role in salt adaptation.

*LOC_Os04g37570* (*OsAP37*) encodes a caspase-like aspartic protease previously validated as a direct *OsNAC2* target in salt-induced programmed cell death (Mao et al., 2018). In this network, *OsAP37* combined high connectivity with substantial induction, suggesting a role in linking PCD regulation to the broader co-expression network. *LOC_Os03g12820* (*OsSRO1c*), a direct SNAC1 target (You et al., 2013a), maintained high absolute expression across both control and salt stress conditions, reaching the highest TPM value among all 20 hub genes under severe salt stress. This expression pattern is consistent with its established role in stomatal closure and H_2_O_2_ regulation under osmotic stress (You et al., 2013a), and aligns with the drought- and cold-sensitive phenotypes reported for its loss-of-function mutants (You et al., 2013a; b). *LOC_Os08g16910* encodes a receptor-like cytoplasmic kinase (RLCK) with both high connectivity and strong induction. By analogy to the rice RLCK STRK1, which phosphorylates catalase C to enhance H_2_O_2_ decomposition (Zhou et al., 2018), *LOC_Os08g16910* may similarly link stress perception to H_2_O_2_ homeostasis, potentially intersecting with MAPK cascades. Functional validation is required to test this hypothesis.

The 15-node hyperosmotic salinity response subnetwork displayed a distributed architecture with multiple interconnected hubs and no dominant central node. *OsDREB1A*, despite its highest global connectivity, was peripherally positioned within this function-specific subnetwork, further supporting a multi-gene coordination model for hyperosmotic salinity adaptation in leaf tissue.

### 4.3 Conservation across varieties and implications for breeding

PCA showed that the transcriptomic landscape was shaped jointly by varietal identity and treatment intensity: treatment groups were ordered along PC1 within each variety, and LJ 11 was consistently separated from the other two varieties along this axis. Despite this divergence, the brown module emerged as the most salt-responsive module across all genetic backgrounds. The low cumulative variance explained by the first two PCs (13.9%) in this experiment reflects the multi-factorial nature of the data: three distinct genetic backgrounds, three treatment levels, and the high dimensionality of transcriptomic responses. Varietal-specific transcriptional variation likely reflects distinct baseline expression landscapes shaped by genetic background. The brown module nevertheless showed dose-dependent activation under progressive salt stress (CK→S5→S8), consistent across all varieties. This conservation aligns with the view that salt stress triggers evolutionarily conserved signaling pathways (Zhu, 2002), comprising ABA-mediated osmotic signaling, phenylpropanoid-based structural defense, and metabolic remodeling. Cross-species evidence supports this: in Arabidopsis, a substantial proportion of salt-induced genes respond to osmotic and cold stresses in a similar manner, forming a shared core stress transcriptome across stimuli and genetic backgrounds (Kreps et al., 2002).

Comparative transcriptome analysis of salt-stressed indica and japonica rice has shown that most salt-responsive DEGs are genotype-specific. The shared DEGs were enriched in abiotic-stress-related GO terms such as plant-type cell wall organization and hydrogen peroxide metabolic process (Kong et al., 2021). The salt-tolerant japonica genotype additionally recruited hormone signaling and transport pathways (Kong et al., 2021; Wang et al., 2023). Notably, the number of shared DEGs in that study increased with stress duration, paralleling the dose-dependent increase in shared DEGs across the three varieties in this study. The 205 and 1,093 shared DEGs identified here can be regarded as a candidate core japonica salt-response set that can be cross-referenced against published datasets to prioritize broadly applicable breeding targets.

The brown module was identified from leaf tissue, whereas canonical salt tolerance mechanisms such as SOS-mediated Na^+^ exclusion and HKT1;5-driven xylem Na^+^ retrieval operate primarily in roots (Zhu, 2002). Root apoplastic barriers further block Na^+^ transport to shoots (Krishnamurthy et al., 2011). These findings suggest a conserved leaf-level transcriptional program that operates alongside canonical root-based mechanisms. Brown module hub genes — notably *OsDREB1A* and *OsAP37* — represent candidate targets for enhancing salinity tolerance through leaf-level adaptive engineering. However, inter-varietal divergence along PC1 indicates that genetic background measurably shapes the transcriptomic landscape, which may constrain the efficacy of these candidates across germplasm. Validation in diverse varieties is thus required prior to breeding deployment.

### 4.4 Limitations of the experimental design and a working model

Several features of the experimental design may explain why the results converged on a largely conserved co-expression network rather than variety- or dose-specific modules. The three varieties are all japonica lines with basal salt tolerance, and their phenotypic divergence under the tested conditions was modest (**Table 1**). Only one developmental stage (three-leaf seedlings), one tissue (leaf) and one sampling time point (10 days of treatment) were analyzed. Genotype- or dose-dependent responses at other stages, tissues or time points were therefore not captured. The network was constructed from the combined 27-sample dataset, which favors co-expression structure shared across varieties. With nine samples per variety, formal module preservation testing and separate per-variety networks lacked statistical power (Langfelder et al., 2011). Phenotyping covered only three indices. Biomass, root traits and physiological measurements such as Na^+^ content, chlorophyll content and antioxidant enzyme activities were not collected. The predominance of shared responses may nevertheless be genuine. In a comparative transcriptome study of indica and japonica rice, shared salt-responsive DEGs increased with stress duration (Kong et al., 2021), paralleling the dose-dependent increase observed here (205 under S5 versus 1,093 under S8).

On this basis, we propose a working model for the leaf-level salt stress response in japonica rice (**Figure 7**). Osmotic and ionic signals imposed by salt stress may be relayed through the MAPK and ABA signaling components enriched in the brown module. These signals may activate transcription factors and downstream effectors involved in osmotic adjustment, phenylpropanoid-based cell wall reinforcement and glycolytic ATP production for ion transport. The basal metabolic processes of the green and blue modules may provide housekeeping support. Within this program, intramodular connectivity and transcriptional induction are only partially coupled. Highly connected genes such as *OsDREB1A* are positioned to coordinate the network, whereas strongly induced genes such as *LOC_Os10g35080* may act as effectors. Because pathway enrichment indicates statistical association rather than direct functional evidence, this model remains a working hypothesis awaiting experimental validation. Connectivity and inducibility should therefore be considered jointly when prioritizing candidate genes for functional validation and molecular breeding.

**Figure 7:**
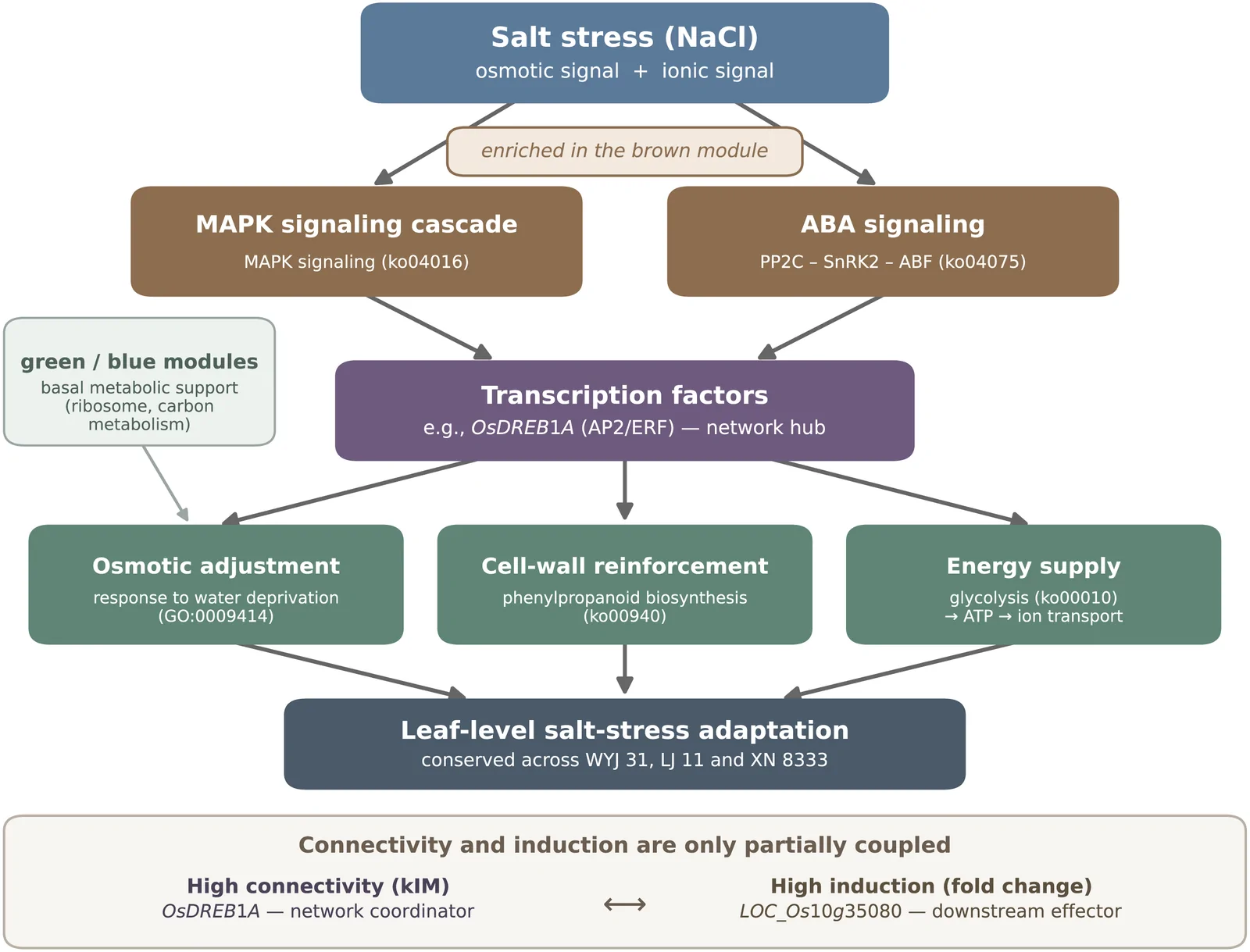
Working model of the leaf-level salt stress response in japonica rice.

## 5 Conclusion

This study used RNA-Seq and WGCNA to characterize salt stress responses in three japonica rice varieties. Despite substantial inter-varietal transcriptional divergence, the brown module was conserved across all varieties and responded dose-dependently to progressive salt stress. This module appears to coordinate adaptation through ABA-mediated osmotic signaling, phenylpropanoid-based cell wall reinforcement, and glycolytic ATP supply for ion transport. Hub genes including *OsDREB1A*, *OsAP37*, *OsSRO1c*, and *LOC_Os10g35080* represent candidates for functional validation. Among these, *LOC_Os10g35080* is uncharacterized and a priority for CRISPR-based validation. Whether it contributes to Na^+^/K^+^ homeostasis, H_2_O_2_ scavenging, or lignin deposition remains to be tested.

## Data Availability

The datasets presented in this study can be found in online repositories. The names of the repository/repositories and accession number(s) can be found below: National Genomics Data Center (NGDC), China National Center for Bioinformation, BioProject accession number PRJCA069350, publicly accessible at https://ngdc.cncb.ac.cn/bioproject/browse/PRJCA069350.
