# Supplementary material for: Aconserved salt-stress co-expression network in japonica rice reveals partially dissociated hub gene connectivity and transcriptional induction
Source: Front Plant Sci. 2026 Sep 14;17:1914311. doi: 10.3389/fpls.2026.1914311 (PMC13616709; doi:10.3389/fpls.2026.1914311)
Supplement: Supplementary file 1 [file DataSheet1.pdf]

Supplementary Material

1 Supplementary Tables

1.1 Supplementary Table1 Summary of RNA sequencing data and read mapping statistics for 27 rice samples.

1.2 Supplementary Table 2 Significantly enriched GO biological process (BP) terms for differentially expressed genes (DEGs) in CK vs. S5 comparison.

1.3 Supplementary Table 3 Significantly enriched GO biological process (BP) terms for differentially expressed genes (DEGs) in CK vs. S8 comparison.

1.4 Supplementary Table 4. Significantly enriched KEGG pathways for differentially expressed genes (DEGs) in CK vs. S5 comparison.

1.5 Supplementary Table 5. Significantly enriched KEGG pathways for differentially expressed genes (DEGs) in CK vs. S8 comparison.

1.6 Supplementary Table 6. Pairwise Pearson correlations of module eigengenes (MEs) among the three rice varieties (WYJ 31, LJ 11, and XN 8333) for each WGCNA co-expression module.

1.7 Supplementary Table 7. Significantly enriched KEGG pathways among genes in the green module.

Supplementary Table 8. Significantly enriched KEGG pathways among genes in the blue

2 Supplementary Figures

2.1 Supplementary Figure S1.

Venn diagram of differentially expressed genes (DEGs) among three japonica rice varieties (WYJ 31, LJ 11, and XN 8333) under S5. DEGs were identified by comparing CK with S5 for each variety using DESeq2 ($|\log_2 \text{FoldChange}| \geq 1$, adjusted $P < 0.05$). A total of 205 DEGs were shared across all three varieties.

2.2 Supplementary Figure S2.

Venn diagram of differentially expressed genes (DEGs) among three japonica rice varieties (WYJ 31, LJ 11, and XN 8333) under S8. DEGs were identified by comparing CK with S8 for each variety using DESeq2 ($|\log_2 \text{FoldChange}| \geq 1$, adjusted $P < 0.05$). A total of 1,093 DEGs were shared across all three varieties.

2.3 Supplementary Figure S3.

Standardized module eigengene (ME) profiles across salt treatments (CK, S5, S8) for all identified modules. Error bars indicate standard error. Each panel represents one co-expression module labeled by color.

2.4 Supplementary Figure S4.

Scatter plot of intramodular connectivity (kIM) versus $\log_2$ fold change (S8/CK) for all genes in the brown module. Pearson $r = 0.377$ ($p = 7.20 \times 10^{-40}$); Spearman $\rho = 0.369$ ($p = 3.24 \times 10^{-38}$). The dashed red line indicates the linear regression fit. Top hub genes are labeled with gene identifiers.

2.5 Supplementary Figure S5.

Comparative characterization of the brown, green, and blue co-expression modules. (A) Module eigengene (ME) fold-change (S8/CK) for the three modules. The brown module showed the strongest dose-dependent induction (2.54-fold), compared with the green (1.72-fold) and blue (1.71-fold) modules. (B) Cross-variety eigengene concordance (mean Pearson r) for the brown, green, and blue modules. All three modules exhibited high preservation across genetic backgrounds (mean $r > 0.98$), supporting the robustness of the co-expression structure despite substantial inter-varietal transcriptional divergence. Concordance values for all 32 modules are provided in Supplementary Table S6.
