# Supplementary material for: Aconserved salt-stress co-expression network in japonica rice reveals partially dissociated hub gene connectivity and transcriptional induction
Source: Front Plant Sci. 2026 Sep 14;17:1914311. doi: 10.3389/fpls.2026.1914311 (PMC13616709; doi:10.3389/fpls.2026.1914311)
Supplement: Supplementary file 2 [file Table1.pdf]

Supplementary Table1 Summary of RNA sequencing data and read mapping statistics for 27 rice samples.

Sample	Clean Reads(M)	Clean Bases(G)	Clean Q30(G)	Average Length(bp)	Total Reads(M)	Total Mapped(M)	Total Mapped/Total Reads
CK_11	44.936	6.724	5.998	149.6	44.93	41.518	92.41
CK_12	44.717	6.689	5.99	149.6	44.712	41.028	91.76
CK_13	45.219	6.766	6.032	149.6	45.212	41.631	92.08
S5_11	44.555	6.667	5.882	149.6	44.549	40.282	90.42
S5_12	44.433	6.647	5.845	149.6	44.428	40.936	92.14
S5_13	44.463	6.653	5.872	149.6	44.458	40.347	90.75
S8_11	45.597	6.814	6.071	149.4	45.592	41.528	91.09
S8_12	45.572	6.818	5.992	149.6	45.566	41.747	91.62
S8_13	45.253	6.771	6.008	149.6	45.248	41.536	91.8
CK_21	45.033	6.74	5.921	149.7	45.026	42.833	95.13
CK_22	45.259	6.774	5.923	149.7	45.253	43.069	95.17
CK_23	42.29	6.328	5.588	149.6	42.285	40.109	94.85
S5_21	44.511	6.659	5.857	149.6	44.506	42.283	95
S5_22	44.819	6.707	5.939	149.6	44.813	43.147	96.28
S5_23	45.65	6.828	6.04	149.6	45.644	43.792	95.94
S8_21	47.116	7.046	6.227	149.5	47.11	45.159	95.86
S8_22	45.1	6.743	6	149.5	45.095	42.801	94.91
S8_23	45.848	6.857	6.109	149.5	45.844	43.768	95.47
CK_31	44.524	6.66	5.922	149.6	44.518	41.146	92.43
CK_32	44.827	6.707	5.928	149.6	44.82	41.305	92.16
CK_33	46.151	6.904	6.115	149.6	46.146	42.802	92.75
S5_31	46.101	6.895	6.15	149.6	46.095	42.546	92.3
S5_32	44.738	6.693	5.943	149.6	44.732	41.536	92.86
S5_33	45.048	6.741	5.93	149.6	45.042	41.6	92.36
S8_31	48.903	7.314	6.527	149.6	48.898	45.248	92.53
S8_32	44.77	6.698	5.956	149.6	44.765	41.3	92.26
S8_33	44.431	6.647	5.896	149.6	44.426	40.945	92.16

*Note:CK, control (0 mM NaCl); S5, moderate salt stress (85.6 mM NaCl); S8, severe salt stress (136.9 mM NaCl). The first digit after the underscore indicates rice variety (1= WYJ 31, 2 = LJ 11, 3 = XN 8333); the second digit indicates biological replicate (1-3). Clean Reads, number of reads after quality filtering; Clean Bases, total base count after filtering; Q30, percentage of bases with Phred quality score ≥ 30; Average Length, mean read length; Total Mapped, number of reads mapped to the reference genome; Total Mapped/Total Reads, mapping rate (%).
