# Supplementary material for: Aconserved salt-stress co-expression network in japonica rice reveals partially dissociated hub gene connectivity and transcriptional induction
Source: Front Plant Sci. 2026 Sep 14;17:1914311. doi: 10.3389/fpls.2026.1914311 (PMC13616709; doi:10.3389/fpls.2026.1914311)
Supplement: Supplementary file 3 [file Table2.pdf]

Supplementary Table 2 Significantly enriched GO biological process (BP) terms for differentially expressed genes (DEGs) in CK vs. S5 comparison.

ID	Description	*p*value	*p*.adjust	Count
GO:1901700	response to oxygen-containing compound	1.89E-11	4.46E-08	139
GO:0009628	response to abiotic stimulus	2.04E-10	2.40E-07	154
GO:0009266	response to temperature stimulus	4.97E-09	3.90E-06	63
GO:0097305	response to alcohol	3.97E-08	1.96E-05	58
GO:0009414	response to water deprivation	4.21E-08	1.96E-05	45
GO:0009415	response to water	5.01E-08	1.96E-05	45
GO:0001101	response to acid chemical	3.96E-07	1.33E-04	98
GO:0009737	response to abscisic acid	4.90E-07	1.44E-04	54
GO:0033993	response to lipid	6.40E-07	1.67E-04	69
GO:0005975	carbohydrate metabolic process	7.71E-07	1.81E-04	48
GO:0009961	response to 1-aminocyclopropane-1-carboxylic acid	2.66E-06	5.69E-04	5
GO:0009408	response to heat	5.60E-06	1.10E-03	29
GO:0016052	carbohydrate catabolic process	1.21E-05	2.20E-03	20
GO:0009719	response to endogenous stimulus	1.83E-05	3.07E-03	112
GO:0009725	response to hormone	2.27E-05	3.56E-03	109
GO:0006970	response to osmotic stress	7.93E-05	1.17E-02	52
GO:0009409	response to cold	9.01E-05	1.25E-02	37
GO:0009753	response to jasmonic acid	1.12E-04	1.46E-02	26
GO:0000302	response to reactive oxygen species	1.19E-04	1.47E-02	22
GO:0046434	organophosphate catabolic process	1.44E-04	1.66E-02	12
GO:0006979	response to oxidative stress	1.48E-04	1.66E-02	35
GO:0009636	response to toxic substance	2.04E-04	2.18E-02	29
GO:0009651	response to salt stress	3.80E-04	3.71E-02	44
GO:0046677	response to antibiotic	3.84E-04	3.71E-02	29
GO:0009791	post-embryonic development	3.94E-04	3.71E-02	90
GO:0009755	hormone-mediated signaling pathway	4.23E-04	3.83E-02	40
GO:0005976	polysaccharide metabolic process	4.91E-04	4.04E-02	26
GO:0009644	response to high light intensity	4.94E-04	4.04E-02	13

GO:0009416	response to light stimulus	4.97E-04	4.04E-02	52
GO:0003006	developmental process involved in reproduction	5.55E-04	4.25E-02	86
GO:0009642	response to light intensity	5.60E-04	4.25E-02	18
GO:0009631	cold acclimation	5.84E-04	4.30E-02	7
GO:0048316	seed development	6.19E-04	4.41E-02	48
GO:0016036	cellular response to phosphate starvation	6.97E-04	4.70E-02	9
GO:0009733	response to auxin	7.00E-04	4.70E-02	31
GO:0042335	cuticle development	7.34E-04	4.80E-02	7

*Note: DEGs were identified between CK and S5 treatments. Enrichment analysis was performed using the GO BP annotation category. *p*value, raw *p*-value from hypergeometric test; *p*.adjust, Benjamini – Hochberg adjusted *p*-value; Count, number of DEGs enriched in the corresponding GO term. Significance threshold: adjusted $P < 0.05$.
