# Supplementary material for: Aconserved salt-stress co-expression network in japonica rice reveals partially dissociated hub gene connectivity and transcriptional induction
Source: Front Plant Sci. 2026 Sep 14;17:1914311. doi: 10.3389/fpls.2026.1914311 (PMC13616709; doi:10.3389/fpls.2026.1914311)
Supplement: Supplementary file 4 [file Table3.pdf]

Supplementary Table 3 Significantly enriched GO biological process (BP) terms for differentially expressed genes (DEGs) in CK vs. S8 comparison.

ID	Description	*p*value	*p*.adjust	Count
GO:0009628	response to abiotic stimulus	2.69E-22	8.19E-19	376
GO:1901700	response to oxygen-containing compound	1.68E-14	2.55E-11	302
GO:0009266	response to temperature stimulus	1.86E-13	1.89E-10	136
GO:0009416	response to light stimulus	5.78E-10	4.41E-07	136
GO:0009409	response to cold	1.54E-09	9.37E-07	90
GO:0009314	response to radiation	2.16E-09	1.10E-06	142
GO:0097305	response to alcohol	5.68E-09	2.47E-06	116
GO:0009414	response to water deprivation	6.92E-09	2.63E-06	87
GO:0009415	response to water	9.28E-09	3.14E-06	87
GO:0009725	response to hormone	3.62E-08	1.10E-05	256
GO:0009737	response to abscisic acid	5.91E-08	1.64E-05	110
GO:0009719	response to endogenous stimulus	9.41E-08	2.25E-05	260
GO:0001101	response to acid chemical	9.58E-08	2.25E-05	210
GO:0033993	response to lipid	1.10E-07	2.40E-05	144
GO:0042542	response to hydrogen peroxide	8.71E-07	1.77E-04	28
GO:0000302	response to reactive oxygen species	9.50E-07	1.81E-04	47
GO:0009636	response to toxic substance	1.81E-06	3.25E-04	64
GO:0006970	response to osmotic stress	2.27E-06	3.84E-04	115
GO:0055085	transmembrane transport	2.91E-06	4.66E-04	162
GO:0009738	abscisic acid-activated signaling pathway	8.95E-06	1.36E-03	36
GO:0005975	carbohydrate metabolic process	1.34E-05	1.91E-03	89
GO:0046677	response to antibiotic	1.38E-05	1.91E-03	63
GO:0006979	response to oxidative stress	2.78E-05	3.69E-03	73
GO:0046686	response to cadmium ion	3.69E-05	4.68E-03	64
GO:0009651	response to salt stress	4.87E-05	5.72E-03	96
GO:0080167	response to karrikin	4.91E-05	5.72E-03	32
GO:0006811	monoatomic ion transport	5.07E-05	5.72E-03	123

GO:0097306	cellular response to alcohol	6.19E-05	6.73E-03	41
GO:1901701	cellular response to oxygen-containing compound	7.64E-05	8.02E-03	102
GO:0071215	cellular response to abscisic acid stimulus	1.01E-04	1.03E-02	40
GO:0009408	response to heat	1.05E-04	1.03E-02	50
GO:0034220	monoatomic ion transmembrane transport	1.35E-04	1.24E-02	99
GO:0010038	response to metal ion	1.36E-04	1.24E-02	89
GO:0009791	post-embryonic development	1.39E-04	1.24E-02	203
GO:0009605	response to external stimulus	2.07E-04	1.80E-02	216
GO:0009733	response to auxin	2.21E-04	1.82E-02	65
GO:0048731	system development	2.25E-04	1.82E-02	269
GO:0009755	hormone-mediated signaling pathway	2.27E-04	1.82E-02	84
GO:0032870	cellular response to hormone stimulus	2.45E-04	1.91E-02	99
GO:0009961	response to 1-aminocyclopropane-1-carboxylic acid	2.56E-04	1.95E-02	5
GO:0030001	metal ion transport	2.91E-04	2.16E-02	49
GO:0071396	cellular response to lipid	3.28E-04	2.38E-02	58
GO:0048316	seed development	3.51E-04	2.49E-02	103
GO:2000034	regulation of seed maturation	3.78E-04	2.62E-02	7
GO:0009642	response to light intensity	5.10E-04	3.45E-02	34
GO:0003006	developmental process involved in reproduction	5.37E-04	3.56E-02	191
GO:0071229	cellular response to acid chemical	5.49E-04	3.56E-02	71
GO:0009644	response to high light intensity	5.78E-04	3.67E-02	23
GO:0016052	carbohydrate catabolic process	6.05E-04	3.76E-02	31
GO:0046434	organophosphate catabolic process	6.76E-04	4.00E-02	19
GO:0071495	cellular response to endogenous stimulus	6.82E-04	4.00E-02	103
GO:0009631	cold acclimation	7.06E-04	4.00E-02	11
GO:0071326	cellular response to monosaccharide stimulus	7.22E-04	4.00E-02	7
GO:0071331	cellular response to hexose stimulus	7.22E-04	4.00E-02	7
GO:0071333	cellular response to glucose stimulus	7.22E-04	4.00E-02	7
GO:0048608	reproductive structure development	7.90E-04	4.22E-02	162
GO:0061458	reproductive system development	7.90E-04	4.22E-02	162

*Note: DEGs were identified between CK and S8 treatments. Enrichment analysis was performed using the GO BP annotation category. *p*value, raw *p*-value from hypergeometric test; *p*.adjust, Benjamini − Hochberg adjusted *p*-value; Count, number of DEGs enriched in the corresponding GO term. Significance threshold: adjusted $P < 0.05$.
