# Supplementary material for: Aconserved salt-stress co-expression network in japonica rice reveals partially dissociated hub gene connectivity and transcriptional induction
Source: Front Plant Sci. 2026 Sep 14;17:1914311. doi: 10.3389/fpls.2026.1914311 (PMC13616709; doi:10.3389/fpls.2026.1914311)
Supplement: Supplementary file 5 [file Table4.pdf]

Supplementary Table 4. Significantly enriched KEGG pathways for differentially expressed genes (DEGs) in CK vs. S5 comparison.

ID	Description	*p*value	*p*.adjust	Count
ko00010	Glycolysis / Gluconeogenesis	4.04E-09	1.01E-06	28
ko00500	Starch and sucrose metabolism	2.81E-07	3.52E-05	30
ko00030	Pentose phosphate pathway	1.37E-05	8.74E-04	13
ko00052	Galactose metabolism	1.39E-05	8.74E-04	14
ko04066	HIF-1 signaling pathway	1.16E-04	5.81E-03	13
ko00051	Fructose and mannose metabolism	3.78E-04	1.58E-02	13
ko02000	Transporter overview	6.66E-04	2.39E-02	65
ko00906	Carotenoid biosynthesis	9.84E-04	3.09E-02	8
ko04016	MAPK signaling pathway - plant	1.51E-03	3.87E-02	25
ko00680	Methane metabolism	1.58E-03	3.87E-02	10
ko00620	Pyruvate metabolism	1.70E-03	3.87E-02	13
ko02026	Biofilm formation - Escherichia coli	1.94E-03	4.06E-02	5
ko00902	Monoterpenoid biosynthesis	2.53E-03	4.88E-02	8

*Note: DEGs were identified between control and moderate salt stress treatments across three rice varieties. Enrichment analysis was performed against the KEGG pathway database. ID, KEGG pathway identifier (ko number); *p*value, raw p-value from hypergeometric test; *p*.adjust, Benjamini ‑ Hochberg adjusted *p*-value; Count, number of DEGs enriched in the corresponding KEGG pathway. Significance threshold: adjusted $P < 0.05$.
