# Supplementary material for: Aconserved salt-stress co-expression network in japonica rice reveals partially dissociated hub gene connectivity and transcriptional induction
Source: Front Plant Sci. 2026 Sep 14;17:1914311. doi: 10.3389/fpls.2026.1914311 (PMC13616709; doi:10.3389/fpls.2026.1914311)
Supplement: Supplementary file 6 [file Table5.pdf]

Supplementary Table 5. Significantly enriched KEGG pathways for differentially expressed genes (DEGs) in CK vs. S8 comparison.

ID	Description	*p*value	*p*.adjust	Count
ko02000	Transporter overview (KEGG overview)	1.13E-12	3.17E-10	173
ko00710	Carbon fixation by Calvin cycle	9.92E-08	1.39E-05	30
ko00010	Glycolysis / Gluconeogenesis	2.03E-06	1.90E-04	40
ko00250	Alanine, aspartate and glutamate metabolism	1.22E-05	8.58E-04	22
ko00630	Glyoxylate and dicarboxylate metabolism	2.15E-05	1.21E-03	27
ko03000	Genetic information processing overview	1.14E-04	5.35E-03	108
ko00500	Starch and sucrose metabolism	1.70E-04	6.82E-03	44
ko00051	Fructose and mannose metabolism	4.27E-04	1.46E-02	22
ko00073	Cutin, suberine and wax biosynthesis	4.67E-04	1.46E-02	13
ko00052	Galactose metabolism	7.49E-04	2.07E-02	19
ko04016	MAPK signaling pathway - plant	8.11E-04	2.07E-02	49
ko00030	Pentose phosphate pathway	1.01E-03	2.37E-02	17
ko00680	Methane metabolism	1.50E-03	3.24E-02	17
ko00980	Metabolism of xenobiotics by cytochrome P450	1.88E-03	3.77E-02	22
ko04075	Plant hormone signal transduction	2.10E-03	3.94E-02	77
ko00220	Arginine biosynthesis	2.77E-03	4.57E-02	12
ko00910	Nitrogen metabolism	2.77E-03	4.57E-02	12
ko00480	Glutathione metabolism	3.00E-03	4.68E-02	28
ko00902	Monoterpenoid biosynthesis	3.36E-03	4.97E-02	13

*Note: DEGs were identified between control and moderate salt stress treatments across three rice varieties. Enrichment analysis was performed against the KEGG pathway database. ID, KEGG pathway identifier (ko number); *p*value, raw p-value from hypergeometric test; *p*.adjust, Benjamini − Hochberg adjusted *p*-value; Count, number of DEGs enriched in the corresponding KEGG pathway. Significance threshold: adjusted $P < 0.05$.
