# Supplementary material for: Aconserved salt-stress co-expression network in japonica rice reveals partially dissociated hub gene connectivity and transcriptional induction
Source: Front Plant Sci. 2026 Sep 14;17:1914311. doi: 10.3389/fpls.2026.1914311 (PMC13616709; doi:10.3389/fpls.2026.1914311)
Supplement: Supplementary file 7 [file Table6.pdf]

Supplementary Table 6. Pairwise Pearson correlations of module eigengenes (MEs) among the three rice varieties (WYJ 31, LJ 11, and XN 8333) for each WGCNA co-expression module.

module	n__genes	cor_WYJ 31_LJ 11	cor_WYJ 31_XN 8333	cor_LJ 11_XN 8333	Mean_cor
royalblue	682	0.999	1.000	0.998	0.999
greenyello	836	0.997	1.000	0.998	0.998
green	978	1.000	0.995	0.997	0.997
pink	933	1.000	0.996	0.995	0.997
black	948	0.997	0.999	0.994	0.997
darkred	666	1.000	0.996	0.994	0.996
blue	1287	0.999	0.991	0.996	0.996
red	965	0.991	0.994	1.000	0.995
salmon	788	0.986	0.990	1.000	0.992
darkgreen	664	0.986	0.985	1.000	0.990
paleturquo	87	0.995	0.980	0.995	0.990
brown	1143	0.980	1.000	0.980	0.987
white	470	0.978	0.974	1.000	0.984
lightgreen	717	0.969	0.985	0.997	0.984
darkturqu	664	0.995	0.968	0.989	0.984
grey60	744	0.980	0.971	0.999	0.983
cyan	787	0.970	1.000	0.964	0.978
lightyello	700	0.983	0.956	0.994	0.977
purple	846	0.967	0.955	0.999	0.974
darkgrey	611	0.972	0.947	0.996	0.972
turquoise	1453	0.940	0.995	0.969	0.968
darkorang	473	0.932	0.968	0.993	0.965
steelblue	94	0.981	0.907	0.971	0.953
lightcyan	757	0.906	0.961	0.988	0.952
orange	560	0.895	0.932	0.996	0.941
saddlebro	106	0.752	0.394	0.902	0.683
yellow	1047	-0.850	-0.614	0.937	-0.175

magenta	896	-0.801	-0.862	0.994	-0.223
tan	837	-0.832	-0.844	1.000	-0.225
midnightb	781	-0.989	-0.954	0.988	-0.318
skyblue	147	-1.000	-0.999	0.999	-0.333

Note: module, WGCNA module name (color label); n_genes, number of genes assigned to the module; cor_WY J31_LJ 11, cor_WYJ 31_XN 8333, and cor_LJ11_XN 8333, Pearson correlation coefficients (r) of module eigengenes between the indicated variety pairs across samples; mean_cor, mean of the three pairwise correlation coefficients.
