# Supplementary material for: Aconserved salt-stress co-expression network in japonica rice reveals partially dissociated hub gene connectivity and transcriptional induction
Source: Front Plant Sci. 2026 Sep 14;17:1914311. doi: 10.3389/fpls.2026.1914311 (PMC13616709; doi:10.3389/fpls.2026.1914311)
Supplement: Supplementary file 8 [file Table7.pdf]

Supplementary Table 7. Significantly enriched KEGG pathways among genes in the green module.

ID	Description	*p*value	*p*.adjust	Count
ko04016	MAPK signaling pathway	7.48E-12	2.39E-09	13
ko00904	Diterpenoid biosynthesis	5.15E-10	8.24E-08	13
ko04075	Plant hormone signal transduction	1.22E-08	1.30E-06	11
ko04138	Autophagy	2.06E-07	1.64E-05	11
ko00940	Phenylpropanoid biosynthesis	1.48E-06	9.45E-05	7
ko00500	Starch and sucrose metabolism	2.39E-05	0.001272243	10
ko04140	Autophagy	0.000114751	0.005245776	10
ko04626	Plant-pathogen interaction	0.000154344	0.006173742	8
ko04144	Endocytosis	0.000218039	0.007752513	11
ko03010	Ribosome	0.000254371	0.008139881	12
ko00460	Cyanoamino acid metabolism	0.000464829	0.013522307	5
ko04136	Autophagy	0.000658826	0.017568685	4
ko00982	Drug metabolism	0.000915313	0.021398593	4
ko04666	Fc gamma R-mediated phagocytosis	0.000936188	0.021398593	6
ko00410	beta-Alanine metabolism	0.001283871	0.027389245	5
ko00945	Stilbenoid, diarylheptanoid and gingerol	0.001386591	0.027731819	3
ko00983	Drug metabolism	0.002649388	0.048711275	4
ko00980	Metabolism of xenobiotics by cytochrome P450	0.002961323	0.048711275	4
ko04212	Longevity regulating pathway	0.003099209	0.048711275	5
ko00010	Glycolysis / Gluconeogenesis	0.003284937	0.048711275	7
ko04130	SNARE interactions in vesicular transport	0.003297452	0.048711275	4
ko04922	Glucagon signaling pathway	0.0033489	0.048711275	5
ko04142	Lysosome biogenesis	0.003552568	0.049427029	10

Note: ID, KEGG pathway identifier (ko number); pvalue, raw *p*-value from hypergeometric test; *p*.adjust, Benjamini – Hochberg adjusted *p*-value; Count, number of DEGs enriched in the corresponding KEGG pathway. Significance threshold: adjusted $P < 0.05$.
