# Supplementary material for: Aconserved salt-stress co-expression network in japonica rice reveals partially dissociated hub gene connectivity and transcriptional induction
Source: Front Plant Sci. 2026 Sep 14;17:1914311. doi: 10.3389/fpls.2026.1914311 (PMC13616709; doi:10.3389/fpls.2026.1914311)
Supplement: Supplementary file 9 [file Table8.pdf]

Supplementary Table 8. Significantly enriched KEGG pathways among genes in the blue module.

ID	Description	pvalue	p.adjust	Count
ko03010	Ribosome	9.82E-11	2.95E-08	21
ko03040	Spliceosome	1.02E-06	0.000153109	13
ko03008	Ribosome biogenesis in eukaryotes	2.42E-06	0.000241849	10
ko00500	Starch and sucrose metabolism	4.43E-06	0.000332534	11
ko00904	Diterpenoid biosynthesis	7.19E-06	0.000431223	9
ko05171	Coronavirus disease - COVID-19	1.09E-05	0.000546793	15
ko05417	Lipid and atherosclerosis	3.45E-05	0.00147825	12
ko05014	Amyotrophic lateral sclerosis	5.21E-05	0.001955334	16
ko05418	Fluid shear stress and atherosclerosis	7.60E-05	0.002532621	9
ko03018	RNA degradation	0.000560555	0.016456406	7
ko05132	Salmonella infection	0.000603402	0.016456406	12
ko04075	Plant hormone signal transduction	0.00095039	0.021426837	6
ko05016	Huntington disease	0.000967226	0.021426837	12
ko04626	Plant-pathogen interaction	0.000999919	0.021426837	7
ko05010	Alzheimer disease	0.001160942	0.023218845	14
ko05012	Parkinson disease	0.001450359	0.026593609	11
ko05020	Prion disease	0.001506971	0.026593609	11
ko00250	Alanine, aspartate and glutamate metabolism	0.001651061	0.027517676	6
ko03060	Protein export	0.001859584	0.029361855	5
ko03013	Nucleocytoplasmic transport	0.002001822	0.030027332	7
ko00562	Inositol phosphate metabolism	0.003059016	0.042373843	6
ko04070	Phosphatidylinositol signaling system	0.003107415	0.042373843	5
ko04144	Endocytosis	0.003673145	0.047910583	9

Note: ID, KEGG pathway identifier (ko number); pvalue, raw *p*-value from hypergeometric test; *p*.adjust, Benjamini – Hochberg adjusted *p*-value; Count, number of DEGs enriched in the corresponding KEGG pathway. Significance threshold: adjusted $P < 0.05$.
